# Editorial: Role of Senescence in Neurodegenerative Diseases

**DOI:** 10.3389/fnagi.2022.907670

**Published:** 2022-05-17

**Authors:** Ferit Tüzer, Shankar J. Chinta, Tania Araujo Viel

**Affiliations:** ^1^Department of Neurobiology and Anatomy, Drexel University College of Medicine, Philadelphia, PA, United States; ^2^Department of Biological and Pharmaceutical Sciences, Touro University California, Vallejo, CA, United States; ^3^School of Arts, Sciences and Humanities, University of São Paulo, São Paulo, Brazil

**Keywords:** senescence, neurodegenerative disease, aging, TBI, microglia, astrocytes, neurons

Cellular senescence is permanent cell cycle arrest resulting from persistent DNA damage signaling caused by excessive cell divisions, oxidative or other DNA-damaging stress, excessive growth signaling by oncogenes, or other stresses (Di Micco et al., 2006; Campisi and Robert, 2014). The senescent cells gain a pro-inflammatory secretory phenotype comprising cytokines, chemokines and matrix metalloproteinases, and upregulate anti-apoptotic mechanisms (Coppe et al., 2010; Sah et al., 2021). Thus, while senescence ensures that cells with possibly oncogenic DNA mutations will cease proliferation, these cells will persist and can be detrimental in the long term. The involvement of cellular senescence in neurodegenerative diseases has been shown in many research articles over the past decade. For example, the analysis of human Alzheimer's disease (AD) and Parkinson's disease (PD) brain tissues demonstrated the increased presence of senescent astrocytes increases in both diseases (Bhat et al., 2012; Chinta et al., 2018). Other studies have shown the possible mechanisms by which senescent astrocytes can induce neuronal dysfunction *via* loss of neurotrophic factors, increased release of pro-inflammatory cytokines, or loss of transporters that maintain the synaptic homeostasis (Crowe et al., 2016; Limbad et al., 2020). Removal of senescent cells (glia or neurons) in genetic mouse models or by senolytic drugs that inhibit the Bcl-2/Bcl-xl pro-survival pathways that senescent cells depend on has prevented the onset of disease pathology in AD mouse models (Bussian et al., 2018; Musi et al., 2018; Zhang et al., 2019). This Research Topic brings together new findings on the contribution of senescent cell types in neurodegenerative diseases (ND). It contains two reviews, one summarizing the knowledge on the aging of microglia and advancements in culturing these cells, and another on the presence of multiple senescent cell types and their possible contributions to pathology after traumatic brain injury. In addition, there are two research articles. One demonstrates that astrocyte cellular senescence, which increases with age (Bhat et al., 2012), is toxic to neuronal health. The other investigates the prevalence of functional cognitive disorder, which has almost exclusively been studied in high-education settings. This article focuses on populations with low education levels, with possible implications for low cognitive reserve and increased senescence in the brain.

Yoo et al., present a comprehensive review on the history of isolation and cellular origins of microglia, their functions, and how this is altered with age and ND. First, they discuss previously held notions about age-associated microglia changes and why these changes may not be accurate due to differences in species (animal and human cells). They also discuss a possible mix-up with immune cells that infiltrate as a result of blood-brain barrier dysfunction in neurological conditions, due to a lack of microglia-specific markers in older studies. Then they also discuss recent methodologies of isolating and culturing microglia and their advantages and disadvantages. This information is a valuable resource for researchers willing to explore the aging field or interested in researching age-associated changes in microglia in the context of disease.

Schwab et al., summarize the latest literature on the pathophysiology of mild traumatic brain injury (mTBI) and overlaps with the consequences of cellular senescence. First, the review focuses on the evidence for DNA damage following mTBI and how it may be detrimental. It then goes over how this ties into the induction of senescence and findings of senescence and inflammation in mTBI models and human studies. Finally, it summarizes how multiple molecular manifestations of mTBI, such as increased reactive oxygen species, inflammation, and even protein aggregates such as tau and amyloid-β, can be exacerbated by senescence.

Borelli et al., show that the prevalence of functional cognitive disorder, characterized as mood or cognitive disorder that is not explained by medical or clinical criteria, in a low education setting is similar to that in high education settings. This study is the first to examine this disorder and comorbidities in a low education setting, in which patients may lack the cognitive reserve afforded by education. As the cognitive reserve is protective against the development of cognitive disorders, patients in a low education setting may lack such protective mechanisms and may have higher pathology, such as senescence. This study is an important pioneer for research of cognitive disorders in low education settings.

Morales-Rosales et al. investigate the consequences of astrocyte senescence on neuronal mitochondrial function. Complementing previous findings in the field (Turnquist et al., 2016; Limbad et al., 2020), they show that senescent astrocytes decrease neuronal survival in co-cultured neurons compared to control astrocytes. They further demonstrate the detrimental effects of senescent astrocytes on neurons, such as loss of mitochondrial membrane potential, fusion, mass, and reductive balance in the cell. They also confirm that these changes are relevant in an organismal setting by analyzing young and old rats' brains and showing that old brains have increased senescence and lower oxidative phosphorylation (OXPHOS) protein levels and reductive potential. This study suggests a mechanism by which senescent cells may cause pathology in ND.

Overall, these articles cover senescence in a disease setting (mTBI) and aging and senescence of two different cell types, microglia and astrocytes and contribute to understanding cell senescence involvement in brain aging and ND.

## Author Contributions

FT composed the main outline of the editorial. SC and TV contributed with corrections and additions. All authors approved the submitted version.

## Conflict of Interest

The authors declare that the research was conducted in the absence of any commercial or financial relationships that could be construed as a potential conflict of interest.

## Publisher's Note

All claims expressed in this article are solely those of the authors and do not necessarily represent those of their affiliated organizations, or those of the publisher, the editors and the reviewers. Any product that may be evaluated in this article, or claim that may be made by its manufacturer, is not guaranteed or endorsed by the publisher.

## References

[B1] Bhat R. Crowe E. P. Bitto A. Moh M. Katsetos C. D. Garcia F. U.. (2012). Astrocyte senescence as a component of Alzheimer's disease. PLoS ONE 7, e45069. 10.1371/journal.pone.0045069 22984612 PMC3440417

[B2] Bussian T. J. Aziz A. Meyer C. F. Swenson B. L. van Deursen J. M. Baker J. D. (2018). Clearance of senescent glial cells prevents tau-dependent pathology and cognitive decline. Nature 562, 578–582. 10.1038/s41586-018-0543-y 30232451 PMC6206507

[B3] Campisi J. Robert L. (2014). Cell senescence: role in aging and age-related diseases. Interdiscip. Top. Gerontol. 39, 45–61. 10.1159/000358899 24862014 PMC4211612

[B4] Chinta S. J. Woods G. Demaria M. Rane A. Zou Y. McQuade A.. (2018). Cellular senescence is induced by the environmental neurotoxin paraquat and contributes to neuropathology linked to Parkinson's disease. Cell Rep. 22, 930–940. 10.1016/j.celrep.2017.12.092 29386135 PMC5806534

[B5] Coppe J. P. Desprez P. Y. Krtolica A. Campisi J. (2010). The senescence-associated secretory phenotype: the dark side of tumor suppression. Annu. Rev. Pathol. 5, 99–118. 10.1146/annurev-pathol-121808-102144 20078217 PMC4166495

[B6] Crowe E. P. Tuzer F. Gregory B. D. Donahue G. Gosai S. J. Cohen J.. (2016). Changes in the transcriptome of human astrocytes accompanying oxidative stress-induced senescence. Front. Aging Neurosci. 8, 208. 10.3389/fnagi.2016.00208 27630559 PMC5005348

[B7] Di Micco R. Fumagalli M. Cicalese A. Piccinin S. Gasparini P. Luise C.. (2006). Oncogene-induced senescence is a DNA damage response triggered by DNA hyper-replication. Nature 444, 638–642. 10.1038/nature05327 17136094

[B8] Limbad C. Oron T. R. Alimirah F. Davalos A. R. Tracy T. E. Gan L.. (2020). Astrocyte senescence promotes glutamate toxicity in cortical neurons. PLoS ONE 15, e0227887. 10.1371/journal.pone.0227887 31945125 PMC6964973

[B9] Musi N. Valentine J. M. Sickora K. R. Baeuerle E. Thompson C. S. Shen Q.. (2018). Tau protein aggregation is associated with cellular senescence in the brain. Aging Cell 17, e12840. 10.1111/acel.12840 30126037 PMC6260915

[B10] Sah E. Krishnamurthy S. Ahmidouch M. Y. Gillispie G. J. Milligan C. Orr E. M. (2021). The cellular senescence stress response in post-mitotic brain cells: cell survival at the expense of tissue degeneration. Life 11, 229. 10.3390/life11030229 33799628 PMC7998276

[B11] Turnquist C. Horikawa I. Foran E. Major E. O. Vojtesek B. Lane D. P.. (2016). p53 isoforms regulate astrocyte-mediated neuroprotection and neurodegeneration. Cell Death Differ. 23, 1515–1528. 10.1038/cdd.2016.37 27104929 PMC5072428

[B12] Zhang P. Kishimoto Y. Grammatikakis I. Gottimukkala K. Cutler R. G. Zhang S.. (2019). Senolytic therapy alleviates Abeta-associated oligodendrocyte progenitor cell senescence and cognitive deficits in an Alzheimer's disease model. Nat. Neurosci. 22, 719–728. 10.1038/s41593-019-0372-9 30936558 PMC6605052

